# A comparison of SWATH-MS methods for measurement of residual host cell proteins in adeno-associated virus preparations

**DOI:** 10.3389/fbioe.2025.1579098

**Published:** 2025-05-02

**Authors:** Thomas M. Leibiger, Lie Min, Kelvin H. Lee

**Affiliations:** Department of Chemical and Biomolecular Engineering, University of Delaware, Newark, DE, United States

**Keywords:** host cell proteins (HCPs), adeno-associated virus (AAV), mass spectrometry, SWATH-MS, DIA-NN, data independent acquisition (DIA), liquid chromatography-tandem mass spectrometry (LC-MS/MS)

## Abstract

**Introduction:**

Analysis of residual host cell proteins in adeno-associated virus (AAV) preparations is challenging due to low availability and high complexity of samples. One strategy to address these challenges is through development of improved liquid chromatography-tandem mass spectrometry (LC-MS/MS) methods with greater sensitivity and reduced sample requirement.

**Methods:**

In this work, we compare the performance of four sequential window acquisition of all theoretical fragment ion mass spectra (SWATH-MS) methods for identification and quantitation of residual HCPs in rAAV2, -5, -8, and -9 preparations produced with human embryonic kidney 293 (HEK293) cells and purified using immunoaffinity chromatography. Key SWATH-MS parameters including spectral library construction (data dependent vs. *in silico*), data processing software (DIA-NN vs. Skyline), and mass spectrometer instrument (Sciex TripleTOF 6600 vs. Sciex ZenoTOF 7600) were assessed. Method attributes including sample requirement and processing time, and method outputs including protein and precursor identifications, host cell protein quantitation comparisons across methods, and quantitation coefficients of variance (CV) were considered to help establish a SWATH-MS workflow well-suited for rAAV HCP analytics.

**Results:**

A 78% increase in HCP identifications, 80% reduction in sample requirement, and 70% reduction in instrument runtime was achieved with an *in silico* spectral library, data processing in DIA-NN, and data collection with the Sciex ZenoTOF 7600 instrument (DIA-NN-7600 method) compared to a previously established method using a DDA-derived spectral library, data processing in Skyline, and data collection with the Sciex TripleTOF 6600 instrument (Skyline-DDA-6600 method). Additionally, the DIA-NN-7600 method shows median HCP quantitation CV below 10% for triplicate data acquisitions, and comparable quantitation to other methods for a panel of highly abundant residual HCPs previously identified in rAAV downstream processing.

**Discussion:**

This work highlights a SWATH-MS method with data collection and processing specifically tailored for rAAV residual HCP analysis.

## 1 Introduction

Liquid chromatography-tandem mass spectrometry (LC-MS/MS) tools are crucially important for the in-depth understanding of biological systems, with proteomic applications spanning from the study of cellular mechanisms to drug discovery and biopharmaceutical process development. Various “bottom-up” LC-MS/MS approaches have been developed for detection, quantitation, and monitoring of peptides derived from proteolytic digestion of protein samples (Yates, 1998). Data dependent acquisition (DDA) LC-MS/MS allows for qualitative analysis of diverse protein mixtures using spectrum-based peptide matching to a reference protein database (Zhang et al., 2021). However, due to selection of only the most abundant precursor ions for fragmentation, DDA provides limited resolution on lower-abundances species (Zhang et al., 2013). Targeted acquisition methods such as selected reaction monitoring (SRM) using a triple quadrupole mass spectrometer or parallel reaction monitoring (PRM) with a Orbitrap or Q-TOF instrument can be used for quantitation of selected proteins (Lange et al., 2008; Peterson et al., 2012). For these approaches, pre-defined peptide ions are filtered and fragmented and a select set of fragment ions are measured (SRM), or a full MS/MS spectrum is acquired (PRM). While these targeted approaches can be suitable for providing highly accurate quantitation and monitoring of sample components, they are limited to analysis of several hundred peptides per sample due to the cycle time limitations for monitoring precursor-product ion transitions or collection of full MS/MS spectra for each selected peptide ion (Ronsein et al., 2015; Gotti et al., 2021). Data independent acquisition (DIA) LC-MS/MS allows for sensitive detection and quantitation of peptides by continuous collection of fragment ion spectra for all precursors (Ludwig et al., 2018). The most common DIA methods use sequential precursor isolation and fragmentation windows applied across a broad mass-to-charge (m/z) range allowing for full precursor ion coverage and MS/MS scanning (Venable et al., 2004; Gillet et al., 2012; Zhang et al., 2020). These DIA methods combine the broad coverage of DDA with the reproducible label-free protein quantitation associated with targeted acquisition methods (Ludwig et al., 2018; Gillet et al., 2012; Zhang et al., 2020).

DIA proteomic methods such as sequential window acquisition of all theoretical fragment ion mass spectra (SWATH-MS) are powerful tools within the field of bioprocessing for characterization of residual host cell protein (HCP) content which is a critical quality attribute for biologically-derived therapeutics (Jones et al., 2021; Walker et al., 2017; Carvalho et al., 2024). These tools have been widely applied for analysis of residual HCPs in monoclonal antibody (mAb) production systems to understand underlying mechanisms of impurity persistence (Herman et al., 2023a; Herman et al., 2023b), design improved downstream polishing steps (Ito et al., 2024), and characterize HCP-specific impacts to drug stability and safety (Jones et al., 2021; Wang et al., 2009; Li et al., 2021; Chiu et al., 2017). However, the reporting of specific SWATH-MS methods for HCP characterization of other therapeutic modalities such as recombinant adeno-associated virus (rAAV) vectors is limited. rAAV is the most widely used viral vector for *in vivo* gene therapy applications with over 200 completed or ongoing clinical trials and 7 FDA approved products for the treatment of monogenic diseases (Clinicaltrials.gov, 2025; Clement and Grieger, 2016; Goswami et al., 2019). Despite residual HCP content being a critical quality attribute for rAAV products (Kontogiannis et al., 2024), directly applying previously established SWATH-MS methods for detection and monitoring of residual HCPs in rAAV bioprocesses presents unique challenges. Low rAAV titers necessitate larger-scale process development experiments to isolate enough purified material to meet LC-MS/MS mass injection targets (Wright, 2023). Additionally, rAAV harvest processes result in complex mixtures of both intracellular and secreted HCPs that may persist across one or more downstream unit operations (Vandenberghe et al., 2010). A SWATH-MS workflow suitable for rAAV residual HCP analytics combines broad protein identification and quantitation at a low coefficient of variance (CV) with low sample requirement.

DIA LC-MS/MS performance depends on both acquisition methods and software tools used for data processing. Gotti et al. benchmarked DIA acquisition methods and analysis software including both Skyline and DIA-NN using a panel of 48 human proteins spiked into an *E. coli* proteome background (Gotti et al., 2021). Further studies have used benchmark datasets to understand the impact of spectral library generation and DIA data processing tools on data outputs (Fröhlich et al., 2022; Lou et al., 2023). These evaluations capture the importance of data collection and processing parameters but are often performed using model reference datasets or through analysis of a select number of proteins spiked into a complex heterogeneous background mixture. In this work, SWATH-MS data acquisition and processing methods were explored in the specific context of residual HCP analysis for rAAV vectors with the goals of increasing HCP identifications and reducing sample requirement while yielding consistent protein quantitation and CV across multiple injections. Four rAAV serotypes (rAAV2, -5, -8, and -9) produced using a commercial HEK293 culture system and purified using POROS™ CaptureSelect™ AAVX affinity chromatography were analyzed for residual HCP content using SWATH-MS. The impacts of three key method parameters were evaluated–the spectral library construction, the DIA data processing software, and the mass spectrometer instrument. First, method performance using a spectral library constructed from project-specific DDA data was compared to a spectral library constructed *in silico* from a provided sequence database. Next, peptide and protein coverage and CV of triplicate data acquisitions were compared for data processing in two open-source software suites–Skyline and DIA-NN, the latter of which leverages advanced deep neural network (DNN) machine learning models for protein identification and quantitation. Lastly, the best performing SWATH-MS method run on the Sciex TripleTOF 6600 as determined by number of unique protein identifications across the sample set was transferred to the Sciex ZenoTOF 7600 instrument to examine instrument-specific impacts on HCP identification and quantitation. Overall, these evaluations resulted in a SWATH-MS method for rAAV HCP analysis with increased protein coverage, and reduced sample requirement and instrument runtime.

## 2 Results

**FIGURE 1:**
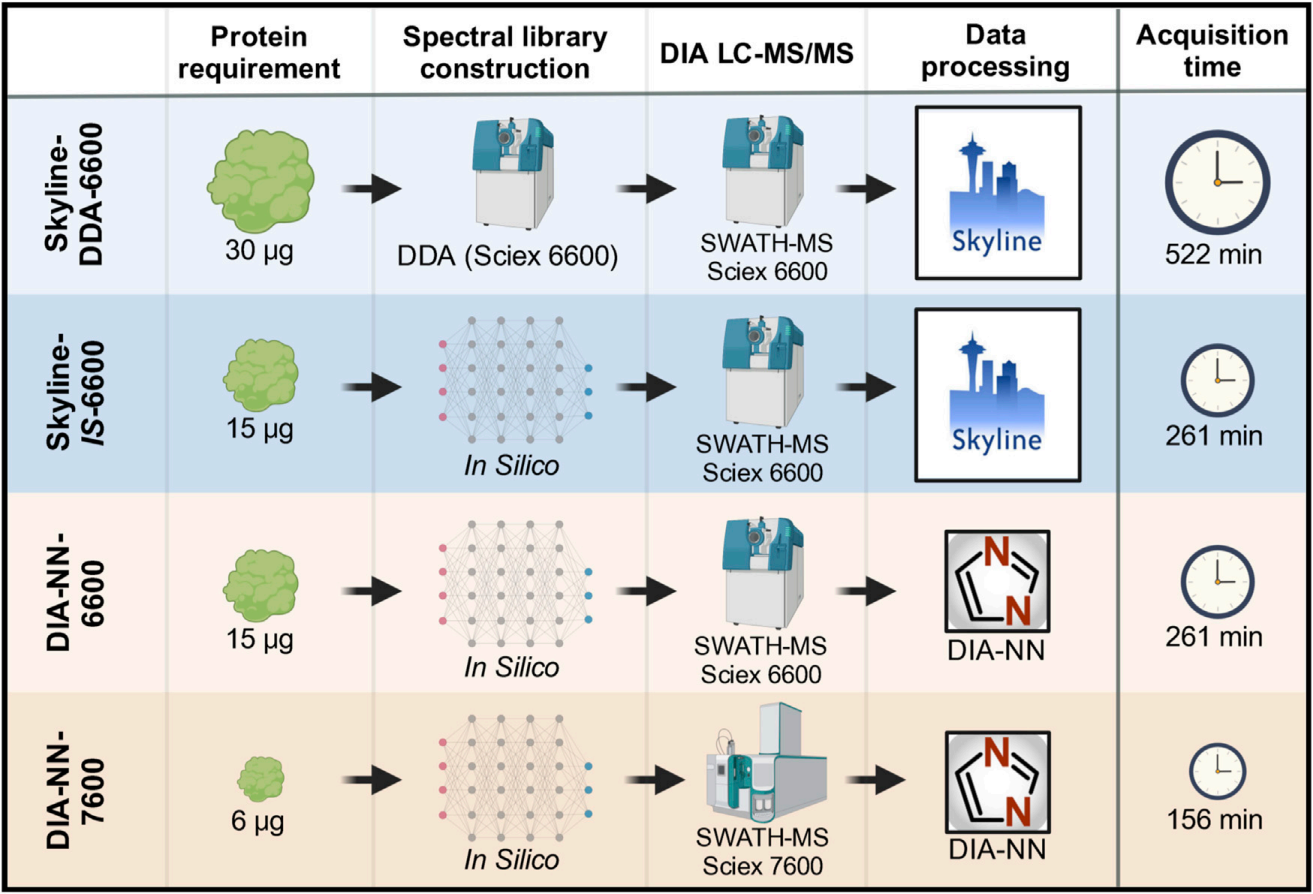
DIA LC-MS/MS data acquisition and analysis overview showing the four workflows tested. Protein requirement and acquisition time are shown for triplicate data acquisition of one sample.

A base-case DIA LC-MS/MS method that was previously established for analysis of residual HCPs in mAb downstream processing uses a spectral library constructed from project-specific DDA data, SWATH-MS data acquisition with a Sciex TripleTOF 6600 instrument, and data processing in Skyline (Skyline-DDA-6600) (Herman et al., 2023a; Herman et al., 2023b; Leibiger et al., 2024a). Protein identification and quantitation outputs for this base-case method were compared to a method using an *in silico* spectral library, a method using DIA-NN data processing software, and a method with data acquisition on a Sciex ZenoTOF 7600 mass spectrometer instrument (Figure 1).

### 2.1 Project-specific DDA vs. *in silico* spectral library (Sciex TripleTOF 6600)

DIA outputs depend heavily on the spectral library composition (Fröhlich et al., 2022; Lou et al., 2023; Zhang et al., 2023). For prior analyses, DDA data based-spectral libraries were built in Skyline with database search results from ProteinPilot software v5.1 (MacLean et al., 2010). Triplicate DDA data was acquired for each sample to feed into spectral library construction which contributed a substantial amount of sample and instrument runtime to the overall DIA workflow. Library-free mode in DIA-NN allows for *in silico* generation of spectral libraries for DIA applications, eliminating the need for DDA data collection and database search (Demichev et al., 2020). SWATH-MS data collected on the Sciex TripleTOF 6600 was processed in Skyline using both a project-specific DDA-derived library and an *in silico* spectral library built using DIA-NN to test library performance.

**FIGURE 2:**
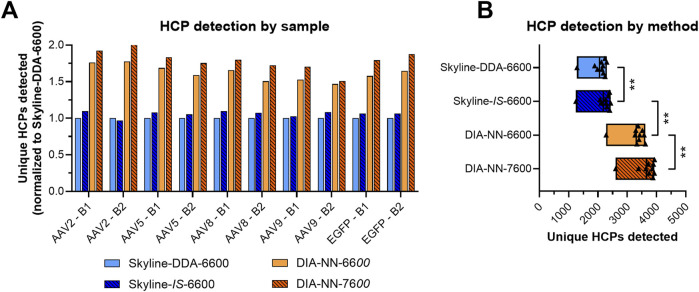
Total HCP identifications for the four different DIA LC-MS/MS workflows **(A)**. Protein identification in triplicate injections was required for inclusion in each method group. Protein identifications averaged across the sample set (*N* = 10) for each method **(B)**. Non-parametric Wilcoxon Signed-Rank statistical tests were applied to determine if the means of paired groups were significantly different. ** indicates statistical significance at p < 0.01.

**FIGURE 3:**
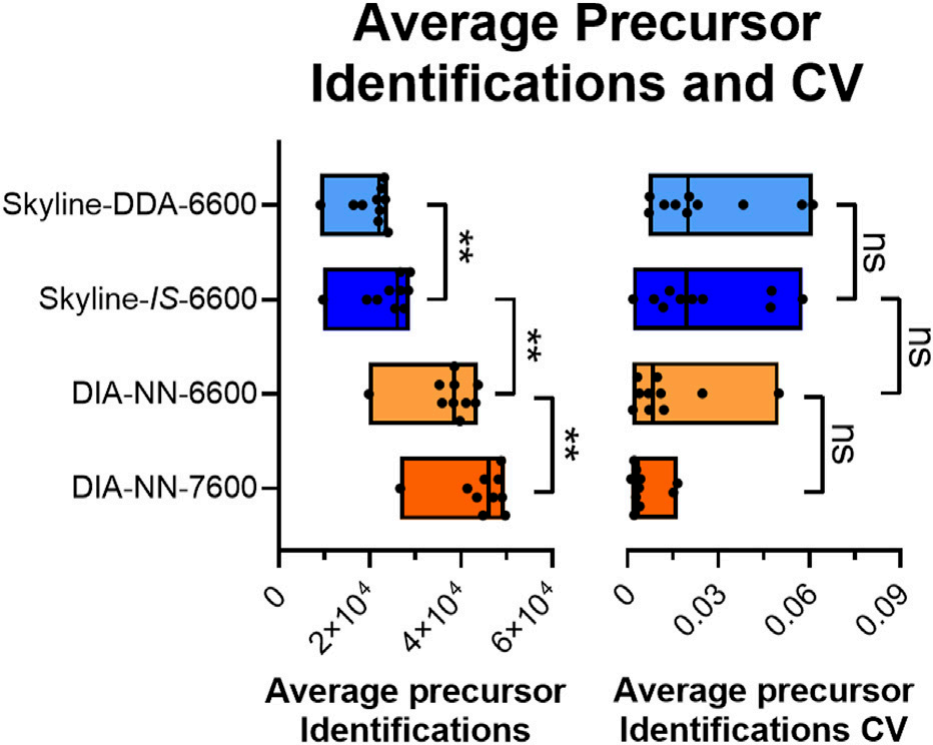
Average precursor identifications and precursor identification CV across triplicate injections for all four SWATH-MS methods across the sample set (*N* = 10). ** indicates statistical significance at p < 0.01. ns indicates that the differences were not statistically significant at a 95% confidence interval.

**FIGURE 4:**
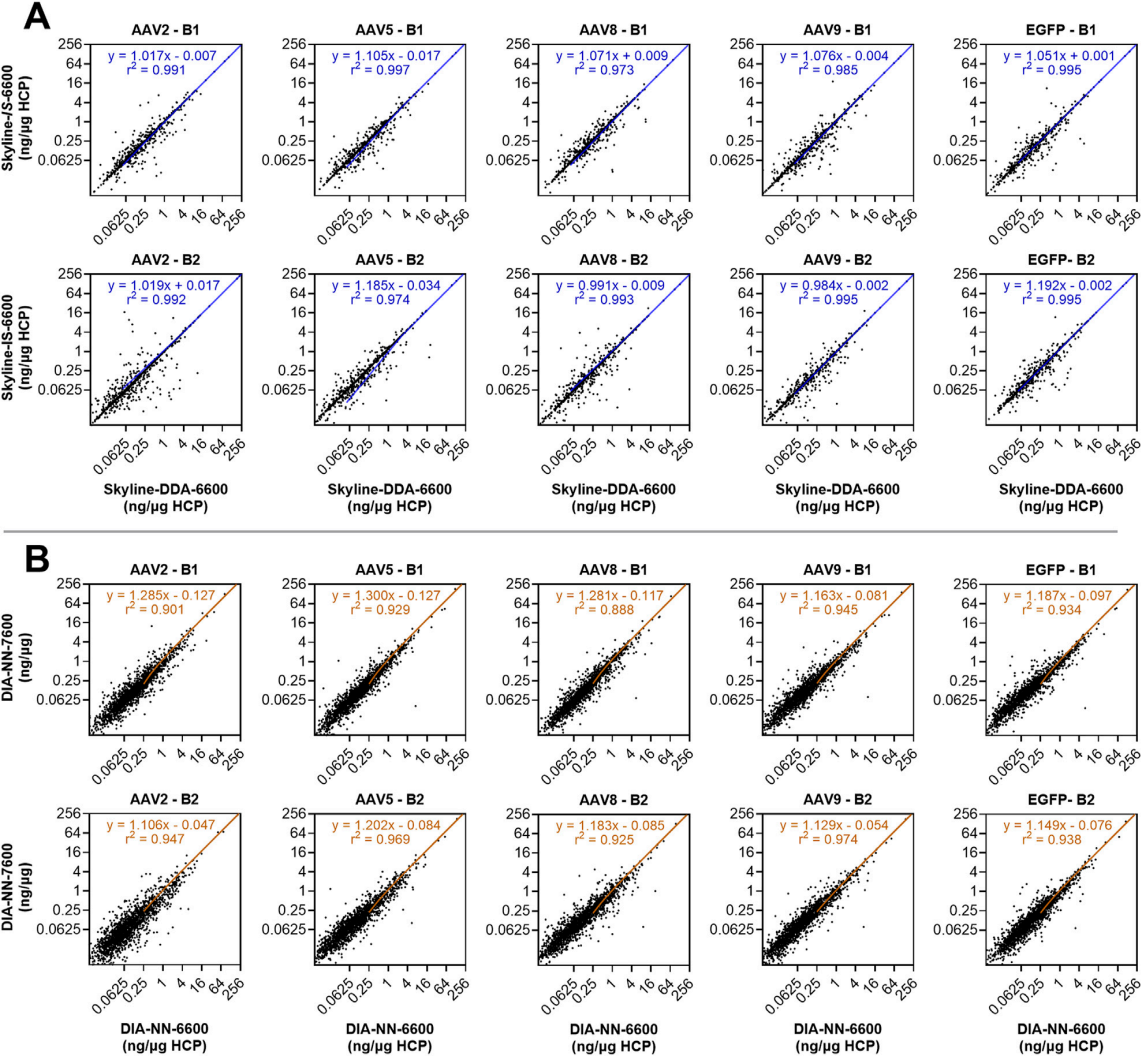
Individual HCP quantitation (ng/µg) for the 961 HCPs commonly quantified with both the DDA and *in silico* spectral libraries in Skyline across all samples (*N* = 10) **(A)**. Individual HCP quantitation (ng/µg) for the 1,758 HCPs commonly quantified with both Sciex instruments using DIA-NN data processing and the *in silico* spectral library across all samples (*N* = 10) **(B)**. Linear regression trendlines of the non-transformed data are shown on each subplot, with equations of fit and goodness of fit *r* ^2^.

**FIGURE 5:**
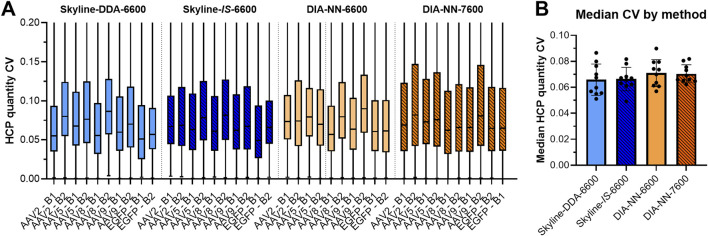
Box plots showing CV values of HCP qunatitation from triplicate data acquisition with each SWATH-MS method **(A)**. Median CV for each method was compared, with each point representing an individual sample (*N* = 10) **(B)**. No statistically significant differences in median HCP quantitation CV were noted across the methods. Box plots are truncated to improve visualization of the 25th–75th percentile region.

Use of the *in silico* spectral library in Skyline (Skyline-*IS*-6600) resulted in a slight increase in protein identifications across the sample set, which was measured to be statistically significant by a non-parametric Wilcoxon Signed-Rank test (p < 0.05) (Figure 2; Supplementary Figure S1). One out of the ten samples analyzed (AAV2 – B2) showed fewer HCP identifications for the *in silico* library (1,246) compared to the DDA library (1,286) causing the conserved protein identifications for the *in silico* library to be slightly below that of the DDA library (Supplementary Figure S1). HCP identifications for all other samples and total unique HCP identifications were higher with the *in silico* spectral library (Figure 2; Supplementary Figure S1). Total precursor identifications trended similarly, with a slight increase for the Skyline-*IS*-6600 method compared to the base case Skyline-DDA-6600 method (Figure 3). There were 961 HCPs commonly identified and quantified in all samples for both DDA and *in silico* spectral library data processing in Skyline (*N* = 10). Normalized protein quantitation (ng of an individual HCP relative to the total HCP amount in µg in the sample) of these species showed excellent agreement between the two methods, with linear regression *r* ^2^ between 0.974 and 0.997 for all samples, and slopes ranging from 0.984–1.192 (Figure 4A; Supplementary Figure S2). There was no statistically significant change to the protein quantitation CV across triplicate injections for the two spectral libraries, with median CV remaining below 10% in all cases (Figure 5). Peptide identification CV also remained consistent for both spectral libraries (Figure 3).

### 2.2 Skyline vs. DIA-NN data processing (*in silico* spectral library, Sciex TripleTOF 6600) – HCP identifications

The DIA-NN software suite houses advanced data processing features which combine peptide- and spectrum-centric approaches with DNNs to discriminate between target and decoy precursors (Demichev et al., 2020). Multiple studies have evaluated the performance of DIA-NN using reference datasets and prepared mixtures of protein standards, broadly demonstrating the superior performance of this software particularly for coverage of low abundance species (Gotti et al., 2021; Fröhlich et al., 2022; Lou et al., 2023).

Data processing with DIA-NN significantly increased protein coverage compared to Skyline, with average protein identifications across the sample set increasing 51.3%, from 2,188 in Skyline (Skyline-*IS*-6600) to 3,310 in DIA-NN (DIA-NN-6600) (Figure 2). Precursor identifications were also significantly higher for the DIA-NN-6600 workflow (Figure 3). There was no observed impact on median HCP quantitation CV for triplicate data acquisition when processing data in DIA-NN compared to Skyline, with median CVs remaining below 10% for all samples (Figure 5). Precursor identification CV also remained consistent for both programs (Figure 3).

### 2.3 DIA-NN data processing with the Sciex ZenoTOF 7600

The DIA-NN-6600 method produced the greatest number of unique protein identifications for runs performed with the Sciex TripleTOF 6600 system. This method was transferred to the Sciex ZenoTOF 7600 instrument (DIA-NN-7600 method), which yielded a further increase in both protein and precursor identifications (Wilcoxon Signed-rank test, p < 0.05). Median HCP quantitation CV was once again unimpacted and remained below 10% for all samples. There were 1,758 HCPs commonly identified and quantified across triplicate injections for all samples using both the DIA-NN-6600 and DIA-NN-7600 methods (*N* = 10). The normalized protein abundances (ng/µg) of these species showed *r* ^2^ of 0.939 for the combined datasets, with a range of 0.888–0.974 across the sample set (Figure 4B; Supplementary Figure S3). However, linear regression slopes were all >1.1 (range 1.106–1.300) indicating that samples re-analyzed with the Sciex ZenoTOF 7600 instrument yielded higher normalized protein abundance outputs despite showing good quantitation linearity with the DIA-NN-6600 data. This variability can be attributed to the separate sample handling required for analysis with the Sciex ZenoTOF 7600 mass spectrometer which included re-measurement of protein concentration, re-digestion, and separate LC-MS/MS data acquisition.

### 2.4 HCP quantitative consistency between DIA LC-MS/MS methods

**FIGURE 6:**
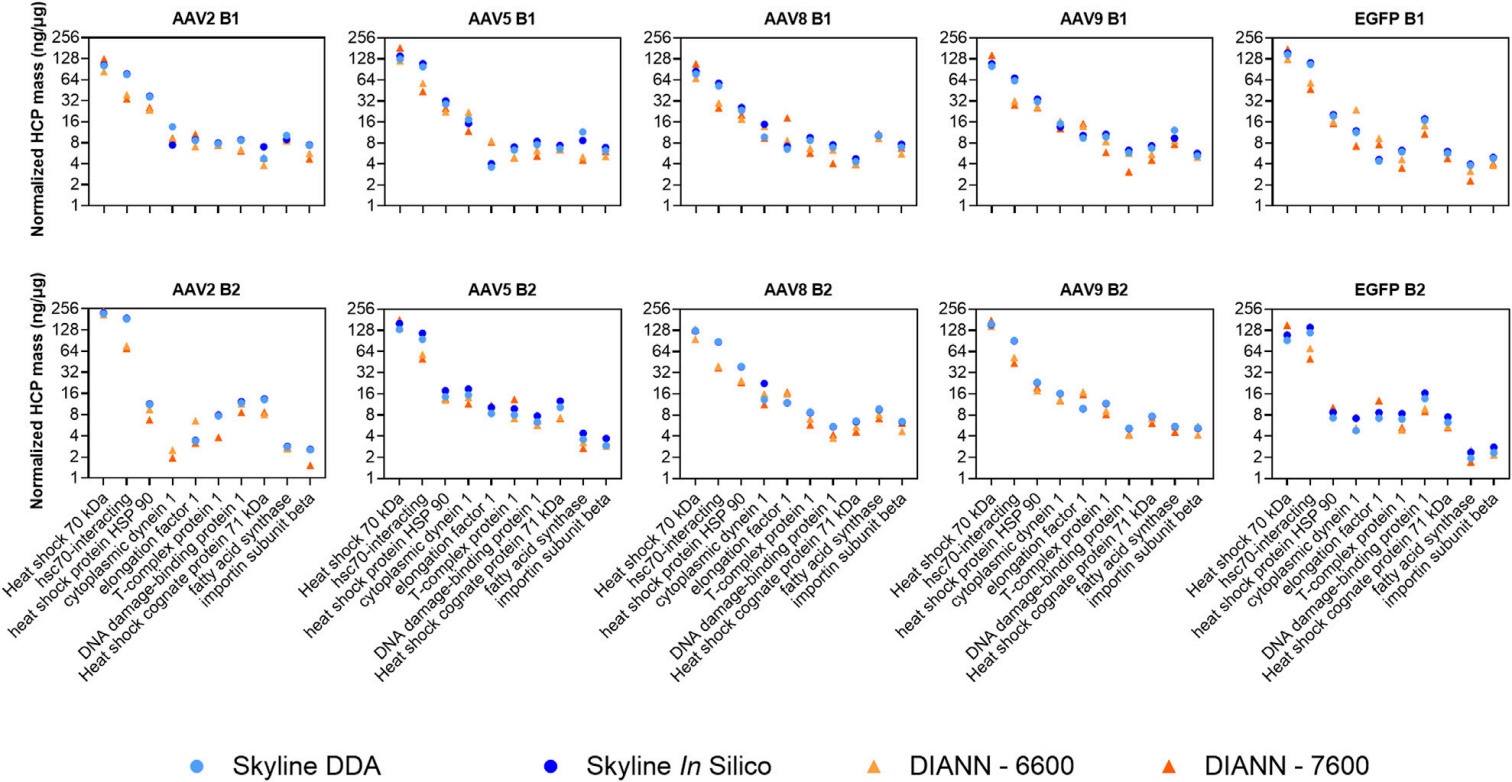
Normalized protein abundance (ng/µg) calculated for each sample across the four SWATH-MS methods. The 10 highest-abundance residual HCPs previously identified for rAAV purification using POROS™ CaptureSelect™ AAVX affinity chromatography were evaluated.

**FIGURE 7:**
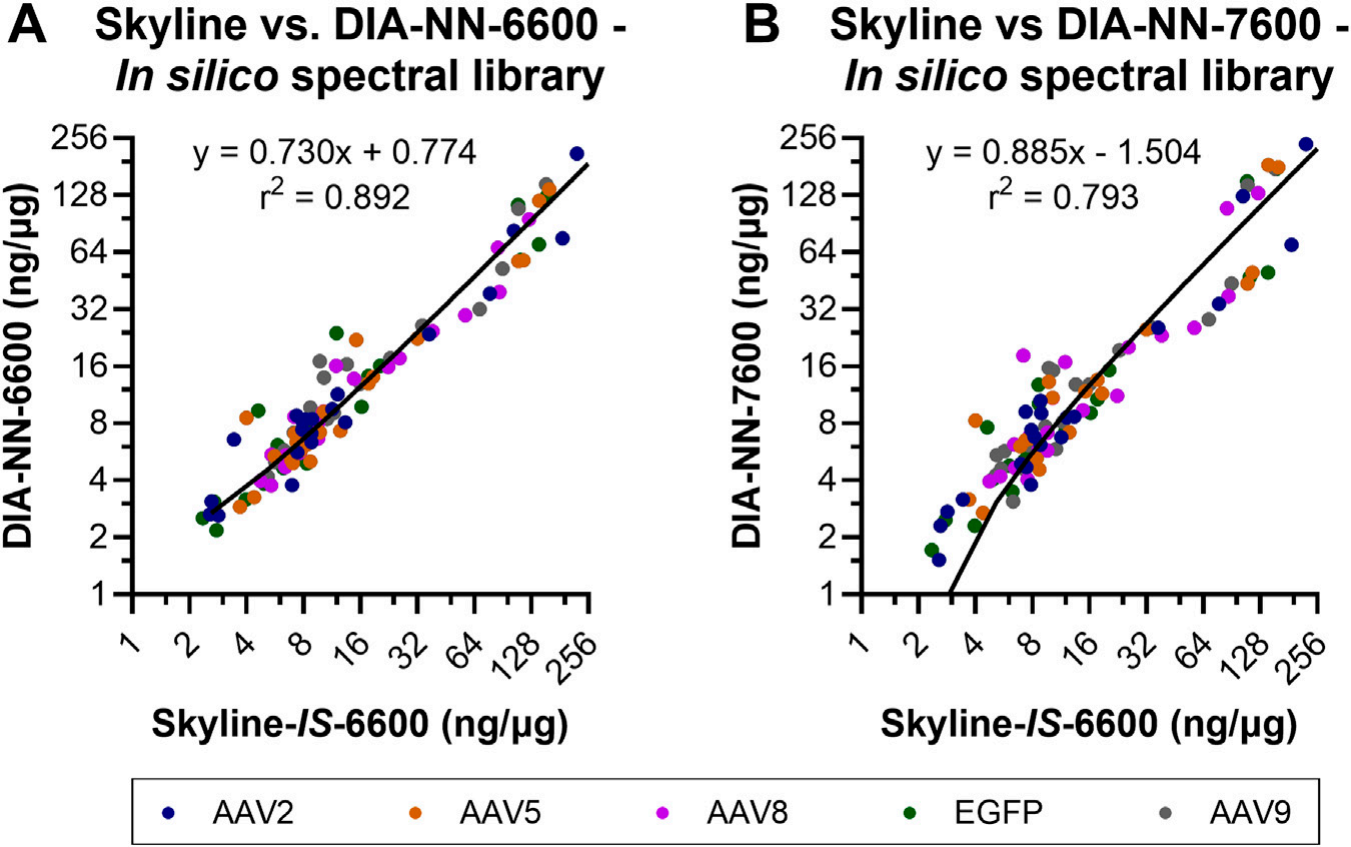
Normalized protein abundance (ng/µg) comparisons between Skyline and DIA-NN for the 10 highest-abundance residual HCPs previously identified (Leibiger et al., 2024a). Skyline-IS-6600 was comapred to DIA-NN-6600 **(A)** and DIA-NN-7600 **(B)**. Data for all samples is plotted together, with color coding designations of rAAV serotypes or EGFP control material. Linear regression was performed across the full datasets. Linear regression trendlines of the non-transformed data are shown on each subplot, with equations of fit and goodness of fit *r* ^2^.

Direct quantitative comparisons of individual HCPs between Skyline and DIA-NN were not performed across the full datasets due to differences in the algorithms governing peak detection, peak integration, and false discovery rate (FDR) calculation. Protein identification outputs for the two software suites were often split across two or more different isoforms that share considerable sequence homology making direct, comprehensive, comparisons challenging. To address this challenge, quantitative outputs for a select set of residual HCPs were manually inspected across the two data processing software. A set of highly abundant conserved HCPs were previously detected within all intermediately purified rAAV2, -5, -8, and -9 preparations analyzed from our production and purification scheme (Leibiger et al., 2024a). The 10 most abundant HCPs across this highly conserved group were compared for the four analysis workflows to determine normalized quantitation (ng/µg) trending of these species (Figure 6). Consistent trends in quantitation were observed across all four methods. Quantitation outputs for these 10 HCPs across the two software suites using the *in silico* spectral library were also compared comprehensively for all samples and showed good linearity between software with *r* ^2^ of 0.892 and 0.793 for DIA-NN-6600 and DIA-NN-7600 compared to Skyline-*IS*-6600, respectively (Figure 7). Slopes of best fit were 0.730 and 0.885 for DIA-NN-6600 and DIA-NN-7600 compared to Skyline-*IS*-6600, respectively, indicating that DIA-NN gives relatively lower normalized HCP abundance compared to Skyline for these HCPs of interest. The lower normalized HCP abundance in DIA-NN can be attributed to dynamic background noise correction, a feature not included in Skyline. Correction factor constants were determined to directly compare protein quantitation based on the mean variability between methods, calculated at 1.250 and 1.365 for DIA-NN-6600 and DIA-NN-7600 vs. Skyline-*IS*-6600, respectively. After applying these correction constants, the median relative difference in protein quantitation between methods was 14.47% for DIA-NN-6600 and 22.57% for DIA-NN-7600 compared to Skyline-*IS*-6600 (26.38% and 32.89%, respectively, without application of correction constants). Despite quantitation agreement for most HCPs, some method-specific trends were also observed. For instance, the normalized abundance (ng/µg) of hsc-70 interacting protein was measured to be approximately 2-fold higher in Skyline compared to DIA-NN (Figure 6).

## 3 Discussion

Process-related impurity retention mechanisms and product impacts have been rigorously explored for mAbs (Herman et al., 2023a; Herman et al., 2023b; Levy et al., 2014; Hogwood et al., 2014), but there have been relatively few studies examining impurity retention for viral vector products (Bracewell et al., 2021; Rumachik et al., 2020; Soni et al., 2024). These studies are challenging to perform for rAAV vectors due to the complex nature and limited availability of samples. The intracellular expression of most rAAV serotypes requires cell lysis at harvest for optimal product recovery which introduces a complex background of cellular impurities into the product stream. Additionally, compared to a high titer mAb bioprocess which can produce 8 g/L, a typical rAAV upstream process may only generate ∼6 mg of packaged vector per liter of cell culture (∼1 × 10^12^ VG/mL culture) making it difficult to meet LC-MS/MS protein mass injection targets with small scale studies that must also support other product-specific analytical workflows (Leibiger et al., 2024a; Szkodny and Lee, 2022). Scaling up current rAAV production systems to better support product and process analytics presents additional complications. rAAV cell culture processes commonly use transient three plasmid transfection of HEK293 cells which is limited by poor mixing of DNA complexes at larger scales and is associated with high-cost consumables such as transfection reagents and plasmid DNA preparations (Lyle et al., 2024). To address these challenges and allow for more rigorous characterizations of residual HCP profiles across rAAV purification, we report a SWATH-MS workflow leveraging *in silico* spectral library construction and DNN data processing with reduced sample requirement and increased sensitivity for low abundance HCPs.

### 3.1 *In silico* spectral library reduces sample requirements and improves protein coverage

Project-specific DDA-based spectral libraries contain high-confidence peptide spectra identifications derived from peptide sequences, precursor and fragment ion data, and retention times. In contrast, *in silico* spectral libraries are composed of computationally-derived peptide fragmentation patterns predicted from a provided protein sequence database (Tiwary et al., 2019). This approach is based on deep learning algorithms and has been used to build hybrid targeted libraries with deeper protein coverage for specific protein families (Lou et al., 2020; Lou et al., 2021), and comprehensive proteome-wide libraries (Demichev et al., 2020; Gessulat et al., 2019). Using an *in silico* spectral library for SWATH-MS of rAAV samples addresses two key challenges in data generation–low sample availability and high sample complexity. Replacing the project-specific DDA spectral library with an *in silico* library constructed from the NCBI:Hu_RefSeqGRCh38 database eliminated the need for DDA data acquisition, reducing sample requirement by 50% while increasing sample throughput and lowering analysis costs. Data processed using the *in silico* library also yielded an increase in identified precursors and proteins across the sample set without impacting individual HCP quantitation trends or CV (Figures 2, 3, 4A). The increase in protein identifications with the *in silico* spectral library is driven by fragmentation pattern prediction of low intensity precursor ions captured in the computationally derived library which may go undetected in DDA data collection. As observed here and previously shown by Rice et al., peptide identifications captured in the DDA but not the *in silico* spectral libraries can occur which may be influenced by charge states of longer ionized peptides with more acidic or basic residues (Rice and Belani, 2022). We observed an increase in mean peptide length for species uniquely detected with the DDA spectral library which aligns with previously reported findings (Rice and Belani, 2022). Using the *in silico* spectral library allows for smaller-scale process development studies or testing of multiple purification conditions from a given material lot which can save time, reduce development costs, and increase sample throughput for rAAV HCP characterization without compromising data quality.

### 3.2 Deep neural network data processing in DIA-NN substantially boosts protein coverage

DIA-NN is a software suite specifically developed for processing DIA proteomics data by leveraging DNNs for differentiation of target and decoy precursor ion patterns using both peptide-centric and spectrum-centric approaches (Demichev et al., 2020). DIA-NN data processing substantially improved HCP identifications for the rAAV samples analyzed, with total detected HCPs increasing 38.8% (2,830 to 3,928, *N* = 8) and universally detected HPCs increasing 88.6% (1,095 to 2,065, *N* = 8) compared to data processed using Skyline (Supplementary Figure S1). The observed variation in data outputs between the two software can be attributed to differences in their computational frameworks. In Skyline, peak detection and integration is primarily driven by direct extraction of chromatographic elements (MacLean et al., 2010). This process includes extraction of m/z intensity and retention time, resampling (i.e., linear interpolation of raw chromatograms), peak detection by local minima, local maxima, and inflection points, peptide modification peak grouping, peptide identification, and peak integration by area under the curve (MacLean et al., 2010; Pino et al., 2020). Peptide identification is based on a static model that uses coefficient-based weighting of peak group features including log intensity, co-elution count, identified count, library intensity correlation, shape score, weighted co-elution, and delta retention time from predicted (Pino et al., 2020). DIA-NN uses a DNN-based machine learning approach for peak detection and integration instead of a relying on static coefficient-based weighting of peak group features (Demichev et al., 2020). Peak group features used for peptide identification are dynamically weighted in DIA-NN based on training data and iterative dataset-specific refinement (Demichev et al., 2020). DIA-NN also employs forward propagating DNNs to discriminate between target and decoy precursors, selecting the best representative precursor-fragment pair for each spectrum while predicting and correcting for interference of co-fragmenting precursors. Because of these features, DIA-NN is particularly well suited for processing proteomics data from complex samples with many co-eluting peptides, making it a preferable software suite for DIA data processing of rAAV samples.

### 3.3 Sciex ZenoTOF 7600 improves HCP detection sensitivity with reduced sample load

Re-digestion and re-collection of SWATH-MS data using the Sciex ZenoTOF 7600 system yielded an additional 10.6% increase in average HCP identifications while reducing sample load by 60% (2 µg injection compared to 5 µg injection with the Sciex TripleTOF 6600 system) (Figure 2). The reduced sample loading with the Sciex ZenoTOF 7600 instrument further addresses sample availability constraints for residual HCP analysis of rAAV samples. This finding is consistent with previously reported SWATH-MS comparisons between the instruments which showed increased protein coverage for the Sciex ZenoTOF 7600 compared to the Sciex TripleTOF 6600 when analyzing a K562 human cell line digest standard at varying sample loading amounts (Wang et al., 2022). Differences in instrument performance result from hardware and software improvements for the newer Sciex ZenoTOF 7600 model, as specified by the manufacturer. The Sciex ZenoTOF 7600 has a faster scan rate leading to enhanced ion picking and a more sensitive ion detector with greater dynamic range compared to the Sciex TripleTOF 6600. The Sciex ZenoTOF 7600’s Zeno trap device can be used to further increase ion utilization and boost signal-to-noise ratio by capturing and releasing ions to the TOF analyzer in synchronized pulses (Wang et al., 2022). The use of this device has been reported to increase precursor and protein identifications potentially allowing for further method sensitivity and sample requirement improvements (Wang et al., 2022).

### 3.4 Conclusion

The base-case Skyline-DDA-6600 workflow requires triplicate data acquisition for DDA library construction with a 5 µg protein injection target, and triplicate DIA data acquisition with another 5 µg protein injection using a Sciex TripleTOF 6600 instrument. Transitioning to *in silico* spectral library generation, DIA-NN data processing, and data acquisition using a Sciex ZenoTOF 7600 instrument provided notable advantages in sample requirement, protein coverage, and instrument runtime. The DIA-NN-7600 method gave a 77.0% average increase in HCP identifications across the full sample set (range 50.7%–102.6%, *N* = 10), an 80% reduction in sample requirement (30 μg–6 µg), and a 70% reduction in instrument runtime (87 min DDA and SWATH-MS to 52 min SWATH-MS only, per injection) compared to the Skyline-DDA-6600 method. These method improvements enhance the ability to study residual HCPs in rAAV bioprocessing, ultimately allowing for more comprehensive process development and vector characterization.

## 4 Materials and methods

### 4.1 rAAV production

rAAV vectors were produced by transient three plasmid transfection of suspension HEK293 cells. Plasmid DNA was complexed with FectoVIR-AAV^®^ chemical transfection reagent, and DNA complexes were added to HEK293 cells at a density of 2.5 × 10^6^ cells/mL dropwise while gently swirling flasks. The cell line present in this study was obtained from Thermo Fisher Scientific. EXPI293^®^ (Thermo Fisher Scientific) cells were exchanged into fresh EXPI293^®^ Expression Media (Thermo Fisher Scientific) immediately prior to transfection. Detailed transfection parameters are summarized in Supplementary Table S1. Plasmid information and acknowledgements are listed in Supplementary Table S2. For each rAAV serotype (rAAV2, -5, -8, and -9) 2 × 1 L shake flasks were transfected for each biological replicate, giving a total of 4 × 1 L shake flasks per serotype. After harvest, the 2 × 1 L flasks of each biological replicate were pooled together to give a total of eight rAAV-containing harvest lots. An additional 1 L flask for each repeated transfection lot was generated with only the pEGFP plasmid delivered to cells to produce ‘control’ EGFP material containing no AAV capsids. For each biological replicate production, a total of 9 × 1 L shake flasks were transfected (two for each rAAV serotype, and one for control EGFP lot). Biological replicate transfections were performed with independent plasmid lots, cell vial thaws, and transfection reagent lots to account for process variability. After pooling replicate flasks, 10 material lots in total were produced, *N* = 2 for rAAV2, -5, -8, -9, and EGFP.

### 4.2 rAAV harvest and quantitative real-time PCR

Culture harvest treatment and quantitative real-time PCR (real-time qPCR) were performed as described previously (Leibiger et al., 2024a; Leibiger et al., 2024b). Cultures were harvested by centrifuging for 10 min at 1,000 × g using a 5920 R centrifuge (Eppendorf). Lysates were generated by three freeze/thaw cycles of resuspended cell pellets in Mammalian Lysis Buffer (50 mM Tris-HCl, 150 mM NaCl, 2 mM MgCl_2_, pH 8.5) followed by 25 U/mL Benzonase^®^ Nuclease (Sigma Aldrich) treatment at 37°C for 60 min. Cell debris was removed by centrifuging harvest material at 3,428 × g followed by filtration with 0.22 μm bottle top filter units (Fisher Scientific). Prior to real-time qPCR, harvest lots were treated with DNase (New England Biolabs) by adding 2.5 µL of sample to a mixture of 2.5 µL DNase I, 2.5 µL DNase Buffer, and 17.5 µL molecular biology water and incubating at 37°C for 60 min. Capsid digestion was performed by adding 2.5 µL of 20 mg/mL Proteinase K (Thermo Fisher Scientific) and incubating at 56°C for 90 min. TaqMan™ Fast Advanced Master Mix (Thermo Fisher Scientific) was used for real-time qPCR along with a primer (900 nM) and probe (250 nM) set (IDT) targeting a region within the EGFP transgene as described previously (Leibiger et al., 2024a; Leibiger et al., 2024b).

### 4.3 rAAV purification

Duplicate flasks for each rAAV serotype were pooled to give approximately 50 mL of clarified lysate per rAAV-containing lot. Purification was performed using POROS™ CaptureSelect™ AAVX affinity resin and an AKTA Pure™ (Cytiva) fast protein liquid chromatography system. To account for serotype-dependent vector secretion into the culture supernatant, only purified rAAV isolated from culture lysates was analyzed in this work. For each affinity purification, 10 mL of clarified lysate was 1:1 diluted with Equilibration Buffer (20 mM Tris-HCl, 0.1 M NaCl, pH 7.5) and loaded onto a 1 mL freshly packed column using TRICORN 5/50 (Cytiva) column hardware. Column equilibration was performed with 10 column volumes (ColV) of Equilibration Buffer, followed by loading, washing with 12 ColV of Equilibration Buffer, and elution with 15 ColV of Elution Buffer (0.1 M Glycine-HCl, pH 2.6). Elution pools were collected in 50 mL conical tubes with 1.5 mL (10 v/v%) Neutralization Buffer (1 M Tris-HCl, pH 8.7). Loading, washing, and elution were performed at 2-min residence time (0.5 mL/min).

### 4.4 LC-MS/MS sample preparation

Coomassie Plus (Bradford) Assay Reagent (Thermo Fisher Scientific) was used to measure protein concentration of affinity chromatography elution pools as described previously (Leibiger et al., 2024a). Protein digestion and desalting were performed as described previously (Herman et al., 2023a; Hamaker et al., 2022; Oh et al., 2023). Sample volumes of 100 µL containing 50 µg of measured protein were reduced with 2.5 µL 100 mM TCEP (Thermo Fisher Scientific) and denatured for 1 h at 60°C, followed by alkylation with 5 µL 150 mM iodoacetamide (Sigma Aldrich) in the dark at room temperature for 30 min. Trypsin (Promega) was then added at an enzyme to substrate mass ratio of 1:50 and digestion proceeded at 37°C for 16 h. Trypsin has been shown to have a limited ability to digest AAV viral capsid proteins, and is therefore recommended for protein digestion of rAAV samples to increase relative HCP signal intensity compared to viral capsid proteins (Smith et al., 2023; Guapo et al., 2022). Digestion was stopped by adding 4 µL 20% formic acid (Thermo Fisher Scientific). For samples with a final protein amount of less than 50 μg, digestion was performed with reagent amounts scaled accordingly. Samples were desalted with Omix C18 tips (Agilent), dried with a Speed Vac (Thermo Fisher Scientific) and redissolved in 45 µL 2% acetonitrile with 0.1% formic acid. Samples were spiked with pre-digested ADH (Waters) to a concentration of 5 fmol/μL and retention time calibrants (iRT, Biognosys, Schlieren, Switzerland). Samples equivalent to 5 μg and 2 µg of digested proteins were analyzed in technical triplicate for LC-MS/MS on the Sciex TripleTOF 6600 and Sciex ZenoTOF 7600, respectively.

### 4.5 LC-MS/MS data acquisition on Sciex TripleTOF 6600

LC-MS/MS data acquisition was done as described previously with an Eksigent Nano 425 LC (Sciex) coupled to a Sciex TripleTOF 6600 mass spectrometer (Sciex) and a dual spray source (Herman et al., 2023a; Hamaker et al., 2022; Oh et al., 2023). Samples were injected into a ChromXP C18CL Sciex column (3 mm, 120 Å, 150 mm × 0.3 mm). Separation was performed using water with 0.1% formic acid (mobile phase A) and acetonitrile with 0.1% formic acid (mobile phase B) at a flow rate of 5 μL/min. A separation gradient of 3%–25% mobile phase B over 68 min, 25%–35% mobile phase B over 5 min, and then 35%–80% mobile phase B over 2 min was used followed by column regeneration and re-equilibration. The column temperature was 30°C. SWATH-MS was performed with a survey scan in the mass range of 400–1,200 m/z followed by 64 variable-size isolation windows for MS/MS with accumulation time of 35 ms. Ion source gas one and two were set at 30 and 35 psi, respectively. DDA was performed with a survey scan over a mass range of 400–1,250 m/z and the top 30 precursor ions were selected for fragmentation and MS/MS detection over a mass range of 100–1,500 m/z.

### 4.6 LC-MS/MS data acquisition on sciex ZenoTOF 7600

LC-MS/MS analysis was done on an Acquity UPLC M-class system (Waters) coupled to a Sciex ZenoTOF 7600 mass spectrometer (Sciex) with an OptiFlow source. Samples were injected into a C18 microtrap column (Phenomenex, 10 × 0.3 mm) and washed with 0.1% formic acid for 2 min at 8 μL/min, then eluted to a C18 column (Phenomenex, Kinetex 2.6 mm XB C18, 100 Å, 150 × 0.3 mm). Mobile phase A and B consisted of 0.1% formic acid in water and 0.1% formic acid in acetonitrile, respectively, and a flow rate of 5 μL/min was used. Elution was performed with a program of 3% B for 1 min, 3%–32% B over 44 min, 32%–80% B over 1 min followed by column regeneration and re-equilibration. The column temperature was 30°C. SWATH-MS was performed with a survey scan in the mass range of 400–1,200 m/z followed by 32 variable-size isolation windows for MS/MS with accumulation time of 25 ms. Ion source gas one and two were set at 30 and 60 psi, respectively.

### 4.7 Spectral library construction

The DDA data based-spectral library was built previously (Leibiger et al., 2024a). Project specific triplicate DDA datasets for all samples analyzed were pooled for a combined database search using Paragon Algorithm in Protein Pilot software (v5.1) (MacLean et al., 2010). A local copy of NCBI:Hu_RefSeqGRCh38 database was used, supplemented with ADH, retention time calibrants, and common contaminants. The resulting group file from Protein Pilot search was imported to Skyline (v20.2.0.343, MacCoss Lab, University of Washington) for building a consolidated spectral library using BiblioSpec with peptides identified at 95% confidence score or higher. In total, the spectral library consisted of 84,563 peptide precursors mapped to 3,432 proteins. An *in silico* library was built in DIA-NN (v1.8.1) with the local copy of NCBI:Hu_RefSeqGRCh38 database supplemented with ADH, retention time calibrants, and common contaminants (Demichev et al., 2020).

### 4.8 LC-MS/MS data processing

For the Skyline-DDA-6600 workflow, SWATH-MS data acquired on Sciex TripleTOF 6600 was processed in Skyline (v22.2.0.351) as described previously (Herman et al., 2023a; Hamaker et al., 2022; Oh et al., 2023). Triplicate SWATH-MS datasets were processed together using the Skyline command line interface with the DDA data based spectral library and the following settings: max 1 missed cleavage allowed, variable carbamidomethyl modification of cysteine, the six most intense b- or y-ions at charge 1+ or 2+, from ion 3 to last ion, ion match tolerance 0.05 m/z, five to six fragments picked from library, resolving power of 36,000, retention time tolerance of 4 min. Peaks were selected and scored with mProphet algorithm based on a target decoy approach (Reiter et al., 2011). Peaks detected at a q value above 0.01 were removed from further analysis. Peak areas were exported to MSstats (Choi et al., 2014), in which peak areas were log_2_-transformed, normalized with global standard (ADH) normalization, and the top three features of each protein were summarized using Turkey median polish to obtain protein areas. ADH peak area was calculated based on the following peptides–ANELLINVK, SISIVGSYVGNR, and VVGLSTLPEIYK. Protein areas were normalized to ADH peak area and protein amounts in ng were estimated based on the assumption that all proteins of a given amount of mol generate an equal response.

For the Skyline-*IS*-6600 workflow, the SWATH-MS datasets acquired on TripleTOF 6600 were processed in Skyline the same as the Skyline-6600-DDA library workflow except the *in silico* library was used instead of the DDA-derived spectral library. Peak areas from Skyline were exported to MSstats and integrated for protein areas to calculate protein amounts in ng.

For the DIA-NN-6600 and DIA-NN-7600 workflows, SWATH-MS data acquired on Sciex TripleTOF 6600 and Sciex ZenoTOF 7600, respectively, were processed with DIA-NN (v. 1.8.1) using the *in silico* library with default settings, except for protein interference which was set at isoform IDs. False discovery rate at protein and peptide levels were set to 1%, and match-between-runs was selected. The main report from DIA-NN was imported to MSstats and integrated for protein peak areas to calculate protein amounts in ng.

### 4.9 LC-MS/MS data normalization

HCP amounts in ng were calculated based on ADH response factors and protein molecular weights. Protein quantities obtained for all four methods were normalized for each sample as described previously (Leibiger et al., 2024a). Non-HCP species including AAV capsid proteins, AAV replication proteins, assembly activating protein, EGFP, ADH, and modified trypsin were removed from the analyses. The amounts of remaining HCP species were normalized by dividing the triplicate-averaged individual HCP amounts (ng) by the total HCP amount (ng) of each sample. Fractional HCP content outputs were then multiplied by 1,000 to give HCP mass in ng of individual HCP per µg total HCP. These normalized outputs (ng/µg) were used for all subsequent analyses.

### 4.10 Statistical analysis

Statistical analyses were performed in Graphpad (v. 10.2.3). Due to the small sample size, all statistical comparisons between DIA methods were performed using non-parametric Wilcoxon Signed-Rank tests with p values <0.05 considered significant.

## Data Availability

The mass spectrometry proteomics data have been deposited to the ProteomeXchange Consortium (http://proteomecentral.proteomexchange.org via the jPOST partner repository with the dataset identifier PXD056294 (TripleTOF 6600 data) and PXD060891 (ZenoTOF 7600 data). All other data generated and discussed in this work is available upon reasonable request.
