# Supplementary material for: A comparison of SWATH-MS methods for measurement of residual host cell proteins in adeno-associated virus preparations
Source: Front Bioeng Biotechnol. 2025 May 2;13:1579098. doi: 10.3389/fbioe.2025.1579098 (PMC12081442; doi:10.3389/fbioe.2025.1579098)
Supplement: Supplementary file 2 [file Table1.docx]

Supplementary Material

# Supplementary Data

**Data S1.** Relative protein quantitation (ng) outputs from all SWATH-MS runs (tab 1). Relative protein quantitation outputs for each detected species were normalized in terms of ng HCP/µg total HCP. Normalized data is shown for Skyline (tab 2) and DIA-NN (tab 3) separately.

# Supplementary Figures and Tables

## Supplementary Figures

**Supplementary Figure 1.** Total (All IDs) and conserved HCP identifications for the four SWATH-MS methods tested. Blue bar areas correspond to HCPs identified in all AAV-containing samples (*N* = 10). Grey bar areas correspond to HCPs identified in at least one AAV-containing sample, but not all samples for each method. Stacked bar heights (blue + grey) correspond to HCPs identified in at least one AAV-containing sample for each method.

Supplementary Figure 2. Venn diagram showing the number of universally identified HCPs across all 10 samples for the DDA and in silico spectral libraries (A). To be included in each library condition group, a specific HCP must be identified in all three triplicate SWATH-MS injections in all samples (*N* = 10). Protein quantitation (ng/µg) comparisons for the in silico spectral library and DDA library is shown in aggregate across all samples (961 HCPs across *N* = 10 samples) (B). Venn diagrams showing the number of commonly identified HCP, and HCPs unique to the DDA or in silico spectral library for each serotype condition (C). To be included in each serotype-specific library condition group, a specific HCP must be identified in all three triplicate SWATH-MS injections for each biological duplicate of that condition.

Supplementary Figure 3. Venn diagram showing the number of commonly identified HCPs, and HCPs unique to the Sciex TripleTOF 6600 or Sciex ZenoTOF 7600 instruments using the in silico spectral library with DIA-NN data processing (A). To be included in each library condition group, a specific HCP must be identified in all three triplicate SWATH-MS injections in all samples (*N* = 10). Protein quantitation (ng/µg) comparisons for Sciex TripleTOF 6600 or Sciex ZenoTOF 7600 instruments shown in aggregate across all samples (1758 HCPs across *N* = 10 samples) (B). Venn diagrams showing the number of commonly identified HCP, and HCPs unique to the Sciex TripleTOF 6600 or Sciex ZenoTOF 7600 instrument for each serotype condition (C). To be included in each serotype-specific library condition group, a specific HCP must be identified in all three triplicate SWATH-MS injections for both biological duplicates of that condition.

## Supplementary Tables

Supplementary Table 1. Transfection and cell culture parameters for recombinant adeno-associated virus (rAAV) production*.*

| **Parameter** | **Description or Setpoint** |
| --- | --- |
| DNA mass (µg)/10^6^ cells | 1.57 |
| DNA mass (µg): volume FectoVIR-AAV (µL) | 1.35 |
| pRepCap:pHelper:pEGFP molar ratio | 5:1:0.31 (AAV2) or 1.5:1:1 (AAV5, 8, 9) |
| Complexation medium | Optiplex™ Complexation Buffer (ThermoFisher) |
| Complexation volume (mL) | 10 (5% culture volume) |
| Complexation time (min) | 30 |
| Seeding viable cell density (10^6^ cells/mL) | 2.5 |
| Culture volume (mL) | 200 |
| Culture duration (hr) | 72 |
| Culture vessel | 200 mL shake flask (Corning) |
| Incubator | Informs, 25 mm throw |
| Incubator setpoints | 135 RPM, 37 ºC, 80% RH, 5% CO_2_ |

Supplementary Table 2. Addgene plasmids used for recombinant adeno-associated virus (rAAV) production and acknowledgements.

| **Plasmid** | **Addgene #** | **Acknowledgement** |
| --- | --- | --- |
| pAdDeltaF6  (plasmid) | 112867 | pAdDeltaF6 was a gift from James M. Wilson (Addgene plasmid # 112867 ; http://n2t.net/addgene:112867 ; RRID:Addgene_112867) |
| pAAV-GFP  (plasmid) | 32395 | pAAV-GFP was a gift from John T Gray (Addgene plasmid # 32395 ; http://n2t.net/addgene:32395 ; RRID:Addgene_32395) |
| pAAV2/2  (plasmid) | 104963 | pAAV2/2 was a gift from Melina Fan (Addgene plasmid # 104963 ; http://n2t.net/addgene:104963 ; RRID:Addgene_104963) |
| pAAV2/5  (plasmid) | 104964 | pAAV2/5 was a gift from Melina Fan (Addgene plasmid # 104964 ; http://n2t.net/addgene:104964 ; RRID:Addgene_104964) |
| pAAV2/8  (plasmid) | 112864 | pAAV2/8 was a gift from James M. Wilson (Addgene plasmid # 112864 ; http://n2t.net/addgene:112864 ; RRID:Addgene_112864) |
| pAAV2/9n  (plasmid) | 112865 | pAAV2/9n was a gift from James M. Wilson (Addgene plasmid # 112865 ; http://n2t.net/addgene:112865 ; RRID:Addgene_112865) |
